# Surface Chemistry in Nanoscale Materials

**DOI:** 10.3390/ma2042404

**Published:** 2009-12-16

**Authors:** Jürgen Biener, Arne Wittstock, Theodore F. Baumann, Jörg Weissmüller, Marcus Bäumer, Alex V. Hamza

**Affiliations:** 1 Nanoscale Synthesis and Characterization Laboratory, Lawrence Livermore National Laboratory, Livermore, USA; E-Mails: baumann2@llnl.gov (T.F.B.); hamza1@llnl.gov (A.V.H.); 2 Institut für Angewandte und Physikalische Chemie, Universität Bremen, Bremen, Germany; E-Mails: wittstock1@gmail.com (A.W.); mbaeumer@uni-bremen.de (M.B.); 3 Institut für Nanotechnologie, Karlsruher Institut für Technologie, Karlsruhe, Germany; E-Mail: joerg.weissmueller@kit.edu (J.W.); 4 Technische Physik, Universität des Saarlandes, Saarbrücken, Germany

**Keywords:** nanoporous materials, nanoporous Au, carbon aerogel, surface chemistry, surface stress, atomic layer deposition, catalysis, actuation, hydrogen storage

## Abstract

Although surfaces or, more precisely, the surface atomic and electronic structure, determine the way materials interact with their environment, the influence of surface chemistry on the bulk of the material is generally considered to be small. However, in the case of high surface area materials such as nanoporous solids, surface properties can start to dominate the overall material behavior. This allows one to create new materials with physical and chemical properties that are no longer determined by the bulk material, but by their nanoscale architectures. Here, we discuss several examples, ranging from nanoporous gold to surface engineered carbon aerogels that demonstrate the tuneability of nanoporous solids for sustainable energy applications.

## 1. Introduction

**Figure 1:**
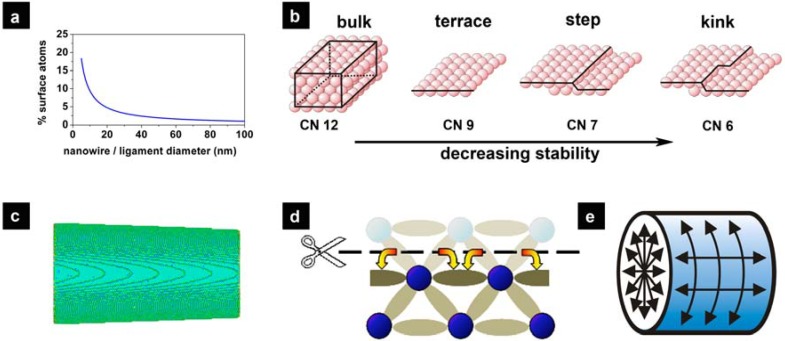
A characteristic property of high-surface area materials is their extremely high surface-to-volume ratio. (a) Typical percentage of surface atoms as a function of the internal length scale. (b) Illustration of terraces and steps for a single crystal surface. (c) Curved surfaces contain a high density of very-low coordination sites such as step edge and kink sites. (d) Surface stress owing to the reduced coordination of surface atoms causing charge transfer to in-plane bonds. (e) Schematic presentation of the origin of the surface stress induced pressure in nanostructured materials which easily can reach the GPa range.

Enrico Fermi reputedly said “God made the solid state. He left the surface to the devil” to describe the fact that surfaces and interfaces are difficult to treat theoretically due to their complex nature. On the other hand, one can exploit this complexity to design tunable interface controlled materials with high surface area such as nanoparticles, nanowires and low-density nanoporous materials. Their surface-to-volume ratio increases with deceasing feature size (Figure 1a), and for small enough feature sizes, their properties are no longer dominated by the bulk of the material but by surface atoms. Based on coordination, one can differentiate between three types of surface atoms which, in the order of decreasing coordination, are terrace atoms, step edge atoms, and kink sites (Figure 1b). Simple geometrical considerations reveal that the curved surfaces, which are typical for most high surface area materials (Figure 1c), are dominated by the more undercoordinated step edge and kink site surface atoms. It is indeed the undercoordination which gives rise to new properties. For example, surface stress is the consequence of an electronic relaxation (Figure 1d) by transferring electronic charge into in-plane bonds [1]. This surface stress induces a pressure in the bulk (Figure 1e) that then further affects the chemical, physical and mechanical properties of the material. For example, theoretical studies have shown that tensile strain can make gold less noble by increasing the ability of gold to bond simple adsorbates more strongly [2,3]. Thus high surface area materials open a new dimension in material design for a multitude of technological important areas, including energy storage and conversion, sensing and catalysis. Beyond morphology, the properties of high surface area materials can be further tuned by surface engineering.

Here we will discuss examples from our own research to illustrate the effect of surface chemistry and surface engineering on the properties of nanoporous materials. Specifically, we will focus on nanoporous gold (np-Au) and carbon aerogels (CAs). Both materials exhibit high porosities (≥70%), and are true three-dimensional materials which can be obtained in the form of millimeter-sized, monolithic samples with well-defined pore-size distributions (including hierarchical porosities) and adjustable densities down to a few atomic percent. We will show that these high surface area materials can be considered as "metamaterials" in a broader sense to describe the fact that their mechanical, chemical, and physical properties are no longer determined by their chemical composition, but are strongly affected by their cellular architecture and their surface chemistry.

## 2. Nanoporous Gold

In the first part of this contribution, we will discuss surface and interface related properties of np-Au. Nanoporous Au is the most studied nanoporous metal in literature (see for example [4,5,6]) due to its easy preparation and its stability in many experimental environments. Nanoporous Au is also a prime example of an interface controlled material: Its stability is controlled by surface chemistry, it is a tunable catalyst material for oxidation reactions, and it has remarkable mechanically properties [7,8,9] that make it a good candidate to exploit surface stress induced macrocopic strain effects for actuator applications. These are the topics that will be discussed in the following sections.

### 2.1. Synthesis

**Figure 2:**
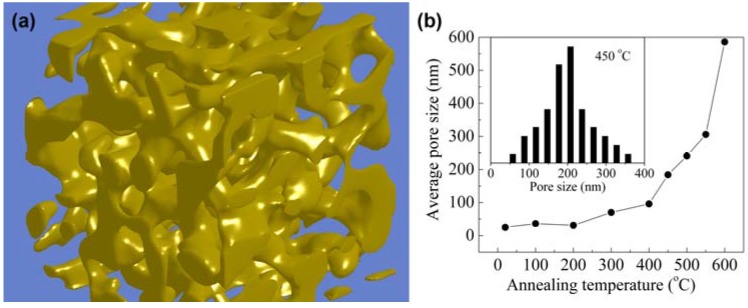
(a) Focused ion beam nanotomography image showing the 3D-structure of bulk nanoporous Au (in collaboration with L. Holzer, Ph. Gasser and B. Münch from EMPA, Switzerland). The ligament size is ~30 nm. (b) Dependence of the average pore width on annealing temperature (Argon atmosphere, 2h). The inset shows the distribution of pore widths after annealing at 450 °C.

Nanoporous Au can be easily prepared in the form of mm-sized monolithic samples by a process called ‘dealloying’. In metallurgy, dealloying is defined as selective corrosion (removal) of the less noble constituent (here Ag) from an alloy (here Ag-Au), and the process was first studied in the context of stress-corrosion cracking [10,11,12,13]. In the case of Ag-Au alloys, the silver can easily be removed by submerging the alloy sample in concentrated nitric acid (so-called “free corrosion”) or by applying an electrochemical driving force in a less corrosive electrolyte (recent reviews can be found in [5,6]). The process works best in a narrow compositional range around Ag_0.7_Au_0.3_ and generates a material with a characteristic three-dimensional bicontinuous nanoporous morphology and a uniform feature size on the nm length scale (Figure 2a). Similar results as those shown in Figure 2a have also been obtained from roughly 100 nm thick thin film samples [14,15]. The pattern formation can be explained by local surface passivation by clustering of Au adatoms in combination with continuous etching of Ag [16]. An amazing feature of this process is that it preserves the grain structure of the original Ag-Au alloy. Thus the grain size in np-Au is typically several orders of magnitude larger than the pore or ligament size [17,18,19]. Materials with grain sizes that are comparable to or smaller than the ligament size have been obtained by dealloying amorphous Au or Pt silicides [20]*.* The specific surface area of np-Au can reach values up to a few ten m^2^/g, and its feature size can be controlled over a wide range, from 4 nm to the micron length scale, by controlling the diffusion kinetics, for example by a simple annealing procedure (Figure 2b) [10,21,22]. Most notably, the material remains self-similar, and the process does not affect the relative density or relative geometry of the material (ligament connectivity or ligament/pore/sample shape) [10,23].

### 2.2. Surface Chemistry and Stability of np-Au

High surface area materials such as np-Au are intrinsically unstable and tend to coarsen with time. The driving force behind coarsening is the Gibbs-Thomson effect, that is, curvature driven growth. On the one hand, this can be used to adjust the feature size of np-Au, for example by annealing. On the other hand, the effect severely limits the stability of np-Au at the lower end of the length scale range. Here, surface chemistry can make an important contribution to enhance the stability of ultra-fine np-Au. It has long been observed that smaller features can be stabilized if dealloying is performed in an electrochemical environment, and slightly more positive dealloying potentials are employed [24,25]. This has been attributed to the onset of Au oxidation that reduces the surface diffusivity of Au adatoms. Unfortunately, the complexity of the dealloying environment makes it extremely difficult to study the role of surface chemistry on the stability of np-Au in more detail.

**Figure 3:**
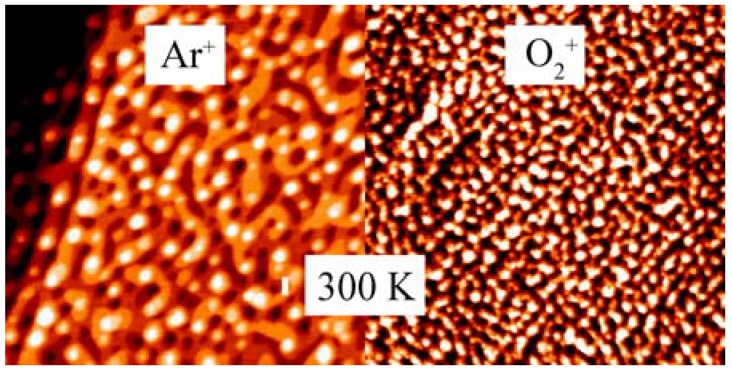
STM images (200 nm × 200 nm) collected from Au(111) surfaces after bombardment with 500 eV argon (left) and oxygen (right) ions (fluence ~3 × 10^15^ ions/cm^2^) at room temperature. Note the smaller length scale of the oxygen-ion-induced surface structure.

Recently, we have demonstrated that ion-bombarded Au single crystal surfaces can be used as a model system to study the effect of adsorbed oxygen on the stability of low-coordinated Au atoms in a well-defined ultra-high vacuum environment [26]. This is possible because dealloying [16,27] and sputtering [28,29] are similar processes on the atomic scale in that they both produce adatoms and vacancies by removing surface atoms, and the evolving morphology is then controlled by surface diffusion and nucleation of both vacancies and adatoms. Figure 3 compares typical STM images obtained from Au(111) surfaces after bombardment with 500 eV Ar^+^ and O_2_^+^ ions, respectively. Both surfaces exhibit the same pit-and-mound morphology, but the characteristic length scale observed after oxygen-ion bombardment is smaller by roughly a factor of two. It can be shown that the smaller length scale of the oxygen-ion induced surface morphology is stabilized by adsorbed oxygen, and that a oxygen coverage of only ~0.3 ML (1 ML = 1.4 × 10^15^ cm^-2^) is sufficient to cause this dramatic effect.

### 2.3. Catalytic Properties of np-Au

**Figure 4:**
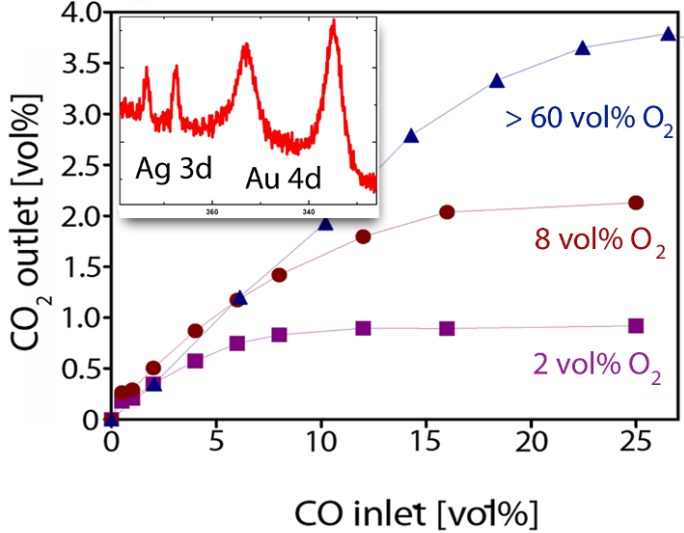
An example demonstrating the high catalytic activity of np-Au for CO oxidation at 20 °C. Shown is the dependence of the CO_2_ concentration produced on the CO concentration supplied for different oxygen partial pressures. Total conversion is observed up to 2 vol % CO, at higher CO concentrations in the feed the conversion becomes limited by mass transport. The measurements were performed in a continuous flow reactor described in detail in ref. [38]. Inset: The amount of Ag present on the ligament surface of np-Au can be quantified by x-ray photoemission spectroscopy (shown are the Ag 3d and Au 4d regions).

The surface chemistry is also important in view of the remarkable catalytic activity of np-Au for CO oxidation (Figure 4) even at low temperatures (up to minus 30 °C) [30,31]. This finding came as a bit of surprise, but demonstrated that new properties can arise from the presence of low-coordination surface sites and specific interface interactions. That finely dispersed gold nanoparticles on suitable oxide supports can show remarkable catalytic activities [32,33,34,35,36,37] is known since the ground breaking work of Haruta, Hutchings, and others [32,37]. But so far it was commonly assumed that both nanoparticle-support interactions and size effects are essential ingredients in making nanodispersed Au reactive.

Nanoporous Au, on the other hand, is an unsupported catalyst and typically exhibits much larger feature sizes than the small diameters of 2–5 nm normally required for supported Au nanoparticle catalysts [32,33,34,35,36], or the thickness of supported bilayer model catalysts studied by Goodman and co-workers [39,40]. Nevertheless, the conversion rates and turn-over-numbers (number of molecules produced per surface atom) observed on np-Au have the same order of magnitude as those obtained from highly active particle systems, demonstrating that np-Au is indeed a highly active oxidation catalyst [38,41]. Furthermore, the catalytic activity of np-Au observed for CO oxidation can last over a period of more than 100 hours demonstrating long-term stability in contrast to many nanoparticle systems.

The common theme of nanoparticle-based Au catalysts and np-Au is that both exhibit curved surfaces with a large fraction of low coordination surface atoms. These sites are crucial for the catalytic activity of gold [36,42] by providing strong adsorption sites for otherwise weakly bound species. For example, density functional theory (DFT) calculations have revealed that the chemisorption energy of CO on an edge site is 3–4 times higher compared to a regular terrace site (−0.6 eV *versus* 0.15 eV) [43]. The activation of molecular oxygen on np-Au, on the other hand, can not be simply explained by the presence of step edge atoms [2,3], and is still subject of discussion. Although low coordination sites such as steps edge sites bind oxygen more strongly than terrace sites [2,3,42,43], the experimentally observed oxygen dissociation rates [44] are still far too low to explain the observed conversion rates on np-Au. In case of supported Au nanoparticles, it is believed that a synergistic interaction with the oxide support is a crucial factor for the activation of oxygen [45]. In contrast, np-Au seems to be best described as an inversely supported gold catalyst [46] or a bimetallic catalyst [31] where the high catalytic activity can be accounted for by the presence of oxidized or metallic Ag residues left over from the dealloying process. This interpretation is supported by recent experiments on CO oxidation [38,47,48] which demonstrate that Ag, even if present only in traces (< 1 at %), plays an active role. Nanoporous Au always contains traces of Ag, and although one can reduce the overall Ag content of np-Au to well below 1 at %, the concentration of Ag at the ligament surface is much higher than its bulk concentration, and typically is in the order of a few percent due to Ag surface segregation [38,48].

**Figure 5:**
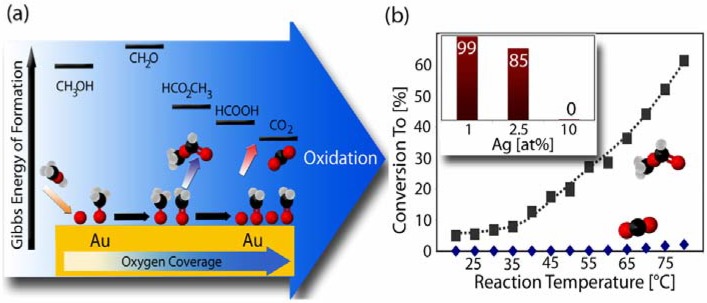
(a) Thermodynamics of oxidation of methanol. Total oxidation to CO_2_ is thermodynamically favored, and selectivity thus requires desorption of desired intermediates prior to further oxidation. (b) Conversion of methanol to methyl formate (grey squares) and CO_2_ (blue diamonds) as a function of the reactor temperature (2 vol % methanol + 1 vol % O_2_). Inset: The selectivity (percent) towards partial oxidation (methyl formate at 80 °C) depends on the amount of residual Ag in np-Au. Reproduced from Ref. [47].

Even more important than its activity is the selectivity of np-Au in partial oxidation reactions. In catalysis, selectivity is defined as the ability to select a specific pathway among others. For example, methanol can be oxidized over np-Au to methyl formate with a selectivity above 97% and a conversion of 60% using molecular oxygen at temperatures below 80 °C (see Figure 5) [47]. Furthermore, the conversion of methanol over np-Au is stable over a period of many days without any sign of degradation (if mild conditions are used), and the turn-over-numbers are compatible with those of supported catalysts even though the latter typically require much harsher conditions [49,50].

According to experiments on single crystal surfaces [51], the initial step of the reaction mechanism is the activation of methanol with surface oxygen. If, however, the surface coverage of atomic oxygen is increased, more and more CO_2_ is formed and the selectivity drops. Thus, abundance of surface oxygen is a key factor for activity and selectivity. In case of np-Au, this is reflected in experiments with varying contents of residual Ag. While samples with small Ag contents show high selectivity for the partial oxidation product (methyl formate), the total oxidation to CO_2_ strongly increases with increasing amount of Ag [47]. This trend confirms again the role of Ag for the activation of molecular oxygen. It is this unique and tuneable surface chemistry which makes np-Au such a fascinating candidate for high end catalytic applications. On the one hand the structure of np-Au provides a large fraction of low coordination surface sites. On the other hand surface-engineering of the material by adjusting the residual Ag enables one to additionally adjust the oxidative power of the material and thus provides a handle to selectivity.

The application of np-Au as large scale catalyst will however also depend on its economical viability. Thus it would be desirable to further increase the surface to volume ratio of np-Au by stabilizing smaller ligaments, and introducing multimodal pore size distributions to overcome mass transport limitations. Towards this end, the low density Au foams made from hollow np-Au shells with densities low as 1.5% [21,52] are very promising candidates. This is comparable to the metal loading levels in supported catalysts.

### 2.4. Charge-Induced Strain and Electrochemical Actuation

Another fascinating aspect of bulk nanoporous materials such as np-Au is that their macroscopic properties can be manipulated by controlling their interfacial properties. The concept of interface controlled nanomaterials was first suggested by Gleiter in the early 1980s [53], and subsequent work concentrated on nanocrystalline solids with grain boundaries as the relevant interfaces [54]. Nanoporous materials, on the other hand, offer two advantages: First, as compared to the bulk, the properties of free surfaces deviate more strongly than those of grain boundaries. This results from the more significant change in atomic coordination at a surface. Second, the surfaces of nanoporous materials with an open porosity can be addressed by external control variables. For example, when a nanoporous metal is used as the electrode in an electrochemical environment, then the surface charge density and adsorbate coverage can be reversibly varied as the function of the electrode potential. An alternative control strategy, which will be discussed in the next chapter, uses the chemical composition of a gas in the pore space as the control parameter. Interface control can be exploited to design nanoscale functional materials with entirely new functionalities. In the following, this concept is exemplified by the macroscopic strain effect observed in nanoporous metal electrodes [55].

**Figure 6:**
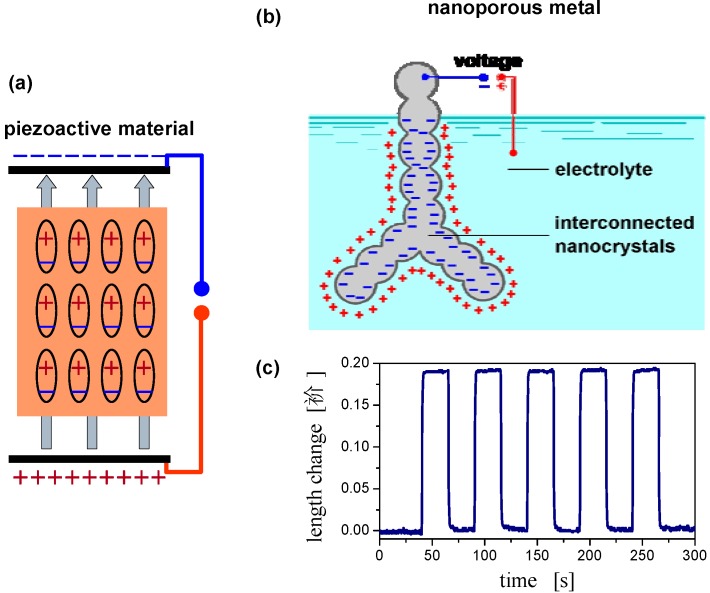
Schematic representation of actuation mechanisms in a conventional piezoceramic (a) and in a nanoporous metal (b). Response of the macroscopic length change to cyclic potential jumps is illustrated by the experimental data in (c). Parts (b) and (c) are reproduced from Refs. [55] and [57], respectively.

As illustrated schematically in Figure 6a, conventional actuator materials, such as piezoceramics, use an electric field as the control parameter. The field, which penetrates the material, prompts a distortion of each crystallographic unit cell and, thereby, a macroscopic strain. Since metals shield electric fields they do not exhibit piezoelectric behavior. Yet, macroscopic nanoporous metal samples exhibit a large reversible strain when the metal is wetted by electrolyte and wired as an electrode in an electrochemical cell [55,56,57]. The origin of this phenomenon is a change in the strength of interatomic bonding at the surface of the metal. Variation of the electrode potential leads to a transfer of charge to the surface. The associated space-charge layer remains confined to the outermost layer of atoms (Figure 6b). Within this region the electron density can be changed by a significant fraction of one electron per surface atom. This gives rise to atomic relaxation and to changes in the electronic structure at the surface [1,58,59]. The combined effect is a change in the lateral attractive or repulsive interaction force between the surface atoms. These modified forces at the surface need to be balanced by stress in the bulk [60], and the strain required to generate this stress is observed as a macroscopic expansion or contraction. Figure 6c displays a typical graph of macroscopic length change *versus* time during potential jumps, see Ref. [57] for details. In fact, the actuation figures of merit of nanoporous metals exhibit values well compatible with more conventional materials [55]. The importance of the surface-induced stress in the ligaments of nanoporous metals is underlined by the finding that the shear component of this stress [60,61] can exceed the theoretical shear strength, leading to a plastic yielding and irreversible densification of the ligament network [18,62].

The central aspects of electrochemical actuation with porous metals can be quantified using a phenomenological approach. The relevant capillary parameter is the surface stress, *f*, the derivate of the surface tension, γ, of the solid with respect to tangential elastic strain. For small values of the surface charge density, *q*, or, in other words, for potentials in the vicinity of the potential of zero charge (*pzc*), *f* varies linearly with *q*. The average pressure in the solid is then also a linear function of *q.* According to the generalized capillary equation for solids [60], the pressure scales with the volume-specific surface area, α, the ratio of surface area to total volume of the solid phase. Even though this phenomenology may appear similar to the familiar Laplace pressure in small droplets [63], a closer look reveals fundamental differences between fluid and solid (Table 1).

**Table 1:** Results for electrocapillary pressure in fluids and solids. The pressure that is exploited in the electrochemical actuation of nanoporous metals is described by the equations and parameters in the column to the right. Note the fundamental difference to the Laplace pressure in fluid droplets, central column. The restrictions on the sign of the individual parameters, indicated in the table, emphasize these differences. Symbols are defined in the main text. For a detailed discussion see Ref. [64].

	Fluid	Solid
**Mechanical balance**	Δ*P* = 2 γ κ	〈*P*〉_V_ = ⅔ α 〈*f*〉_A_
**Geometry parameter**	curvature, κ	Area per volume, α
	**±**	**+**
**Capillary parameter**	surface tension, γ	Surface stress, *f =* dγ/d*e*
	**+**	**±**
**Response to charging**	γ = γ_0_ – *q*^2^ / *c*	*f* = *f*_0_ + ς *q*
	**–**	**±**

The Laplace pressure in fluids scales with the product of surface tension and surface mean curvature, whereas the surface-induced pressure in a solid scales with the product of surface stress and area per volume. The relevant capillary parameters (surface tension or surface stress) as well as the geometry parameters (surface mean curvature, κ, or surface area per volume) are fundamentally different. For instance, γ and α must be positive, whereas *f* and κ can be of either sign. Furthermore, the Lippmann equation [65] requests γ to decrease as a quadratic function of *q*, whereas *f* varies linearly with a slope that can have either sign. Mechanics unambiguously rejects the existence of a Laplace pressure in any solid, and conclusions derived using that concept must therefore be subject to serious doubt. For a critical discussion of common misconceptions in that context, see Ref. [64].

Magnitude as well as microscopic origin of the surface stress-charge response parameter, ς = d*f*/d*q*|*_e_* where *e* denotes a tangential strain variable, are the subject of current research. The simplest scenario is when charging is dominantly capacitive, for instance in dilute solutions of weakly adsorbing ions near the *pzc*. Here, experiments on Au and Pt suggest ς~−2 V [66,67,68]. Experiments also document a trend for anion adsorption to reduce the magnitude of ς [69,70]. Yet, adsorption may still enhance the strain amplitude of nanoporous metals, because the reduction in ς may be over-compensated by an enhanced capacitance and, hence, enhanced value of Δ*f* = ς Δ*q* for a given potential interval. Comparatively large changes in *f* are recorded during the adsorption of oxygen species (usually with ς < 0) and of hydrogen (ς > 0) on clean metal surfaces.

The available evidence suggests that the essential physics behind the charge response of the surface stress is indeed related to the effect of the space-charge on the bonding between the surface metal atoms. Bonds with adsorbed anions may only be relevant in as much as charge is transferred away from the metals and towards the adsorbate, reducing the amount of space charge available for changing the surface stress [66,69,70]. In support of this picture, ab initio electron density functional theory (DFT) computation of ς for a Au(111) surface in the absence of adsorption achieves excellent agreement with experiments [58]. While a detailed understanding of the underlying microscopic processes has yet to emerge, the DFT studies highlight specifically the link between space-charge and a reversible out-of-plane relaxation of the surface layer of atoms [1,59]. For Au(111) the change in the in-plane stress that is quantified by Δ*f* agrees well with estimates based on the transverse elastic response to the concomitant out-of-plane strain [1]. At present, however, theory offers no obvious way of extrapolating the findings for gold to other metals.

The observations on charge-induced strain in metals with oxide-covered surfaces emphasize the decisive role of the atomic and electronic structure of the solid on the stress-charge response. Monolayer-thick metastable oxide layers can be generated by anodic oxidation of Au as well as Pt electrodes. This state of the surface is stable within a sufficiently large potential interval to perform electrochemical actuation experiments. Remarkably, the response parameter ς is here large and positive [56,68]. This contrasts with the negative-valued ς exhibited by the clean metal surfaces in the same potential interval.

### 2.5. Surface Chemistry Induced Macroscopic Strain Effects

In the previous section, we have seen that charge-induced changes of the surface stress in an electrochemical environment can trigger a macroscopic strain response. Recently, this concept has been extended to adsorbate-induced changes of the surface stress at the surface-gas interface [71]. This effect can be used to convert chemical energy directly into a mechanical response thus opening the door to surface-chemistry driven actuator and sensor technologies*.*

**Figure 7:**
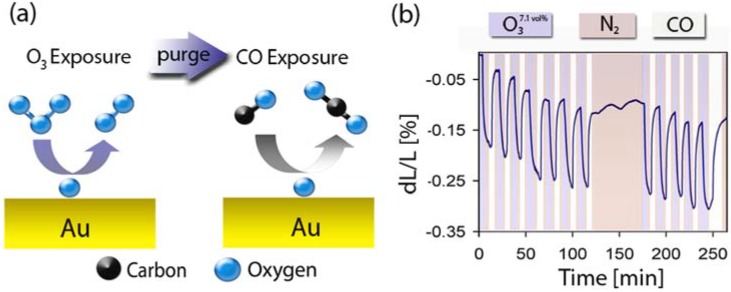
Illustration of surface chemistry driven actuation in nanoporous gold. (a) The surface of np-Au can be switched back and forth between an oxygen-covered and clean state by alternating exposure to ozone (O_3_) and carbon monoxide (CO). (b) Strain *versus* time as the np-Au actuator is alternately exposed to a mixture of ~7 vol % O_3_ in O_2_ and pure CO. Ozone exposure causes contraction, while CO exposure restores the original sample dimension. Reproduced from Ref. [71].

A typical example is shown in Figure 7. Here the surface–chemistry induced strain response of a millimeter-sized np-Au sample is continuously monitored while the sample is alternately exposed to ozone (O_3_) and carbon monoxide (CO). This switches the surface of np-Au back and forth between an oxygen-covered and clean state: The Au surface catalyzed decomposition of ozone leads to an oxygen coverage of up to one monolayer [72], and the CO exposure in the second half cycle completely removes the adsorbed oxygen again by CO_2_ formation [31]. Because the surface stress of the clean Au surface is different from that of the oxygen-covered surface, this results in a macroscopic strain response. In the specific example shown in Figure 7, ozone exposure causes a sample contraction, while CO exposure restores the original sample dimensions. The strain amplitude increases with both ozone concentration and exposure length, and can reach values up to 0.5% (5 μm stroke for a one-mm-long sample). Using molecular dynamics simulations, one can show that the experimentally observed strain level of up to 0.5% requires surface stress changes of a few N/m, consistent with the typical size of surface chemistry induced changes of the surface stress. The example also demonstrates that surface stress and surface chemistry are interconnected, an important observation in terms of catalysis where it has been suggested that tensile strain makes gold less noble.

## 3. Carbon Aerogels

In the second part of this contribution, we will discuss the properties of carbon aerogels (CAs), and how these materials can be tailored for specific applications by controlling their morphology and/or by adding surface functionalities. The design of new porous carbon materials holds technological promise for a variety of applications, including catalysis, hydrogen and energy storage [73,74,75,76,77]. The utility of these materials is derived from their high surface areas, electrically conductive frameworks and chemical stability. CAs are a unique class of porous carbons that possess ultrafine cell sizes, continuous porosities and low mass densities [78]. These properties arise from the aerogel microstructure, a three-dimensional network of interconnected primary carbon particles with diameters that can range from a few nanometers to several microns. In contrast to the metallic bonding in np-Au, CAs are a covalently bound materials which makes them more stable against thermal coarsening. In addition, the graphitic character of the CA surface adds further stability to the structure.

### 3.1. Synthesis

**Figure 8:**
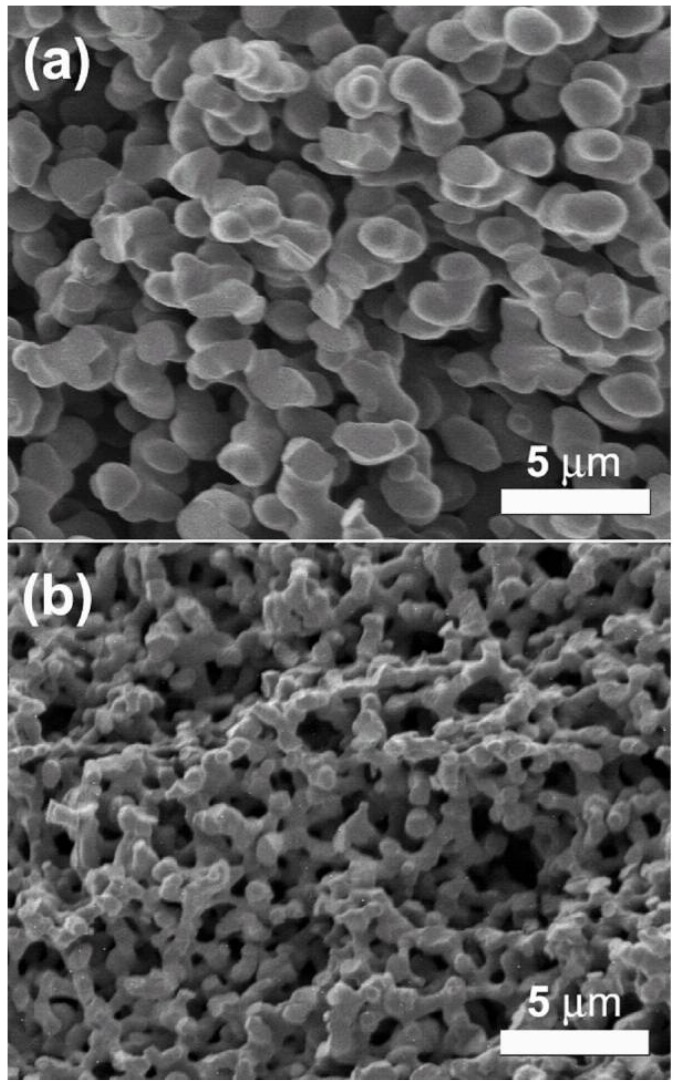
SEM images of (a) the pre-activated CA and (b) an activated CA with surface area of 3,200 m^2^/g.

CAs are prepared through the sol-gel polymerization of organic precursors, such as resorcinol and formaldehyde, in aqueous solution to produce highly cross-linked organic gels that are supercritically dried and subsequently pyrolyzed in an inert atmosphere [79]. Pyrolysis of the organic aerogel then yields a porous carbon network (Figure 8) comprised of both amorphous and graphitic regions. The graphitic domains are typically quite small and contain a significant amount of disorder. Unlike many other porous carbons, CAs can be fabricated in a variety of forms, including monoliths and thin films, a feature that can be advantageous for many applications.

The structure-property relationships of CAs are largely determined by the sol-gel reaction chemistry. Several factors of the polymerization reaction have a significant impact on network formation in these materials. For example, the amount and type of polymerization catalyst used in the sol-gel reaction influences nucleation, growth and interconnectivity of the primary particles that comprise the aerogel framework. The morphology and spatial arrangement of these particles, in turn, determines the bulk physical properties of the CA. For instance, electrical conductivity in CAs occurs through the movement of charge carriers through individual carbon particles and “hopping” of these carriers between adjacent carbon particles [80]. Therefore, charge transport is highly dependent on interconnectivity of the carbon network. Likewise, a number of other bulk properties, such as specific surface area, compressive modulus and thermal conductivity, correlate with the network architecture and, therefore, can be tuned through the reaction chemistry.

Utilizing this flexibility, we recently developed a synthetic approach to fabricate mechanically robust, high surface area CA monoliths for energy storage applications [81]. Our strategy to access high surface areas in monolithic parts involved the thermal activation of a CA material with structural features (particles and pores) on the micrometer scale. The microstructure of traditional CAs, consisting of nanometer-sized carbon particles and tortuous pore structures, can both limit the attainable surface areas through activation and lead to inhomogeneous activation in monolithic samples. By utilizing CAs with larger pore and particle sizes, however, these issues can be mitigated and monolithic CAs with BET surface areas in excess of 3,000 m^2^/g can be prepared. These values are greater than the surface area of a single graphene sheet (2,630 m^2^/g, if both graphene surfaces are taken into account). Presumably, edge termination sites constitute a substantial fraction of the surface area in these activated CAs, as is the case for traditional high surface area activated carbons. Another benefit of this design strategy is that the process yields materials with bimodal porosity (macro- and micropores). Hierarchically porous carbons of this type present a number of advantages over unimodal carbon structures in terms of diffusion efficiency and surface area. Therefore, this approach offers viability to engineer new materials for use as catalyst supports, electrodes, capacitors and sorbent systems.

**Figure 9:**
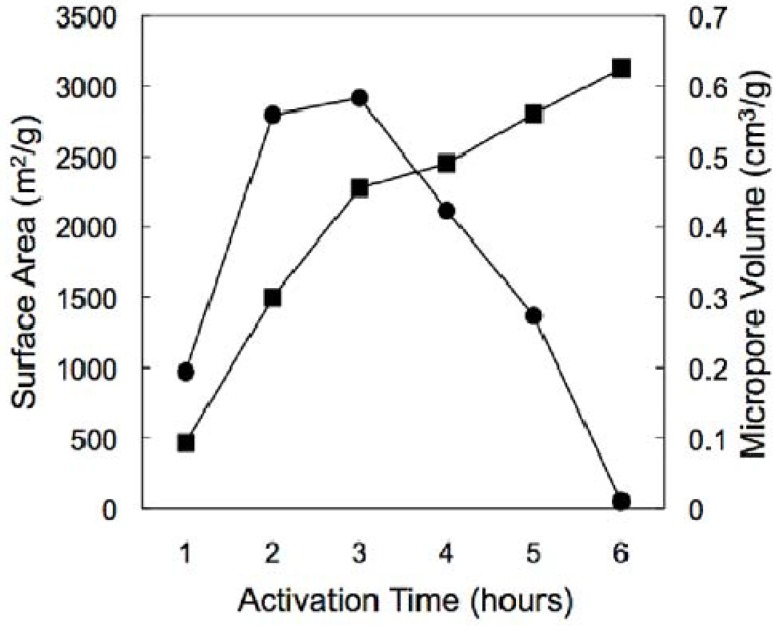
BET surface area (■) and micropore volume (●) for the activated CAs as a function of activation time.

The CA structure used for activation was prepared by the sol-gel polymerization of resorcinol and formaldehyde using acetic acid as the reaction catalyst [81,82,83]. The skeletal structure of this material consists of interconnected micron-sized carbon ligaments that define a continuous macroporous network (shown in Figure 8). These ligaments appear to be made up of spherical primary particles that have fused together during network formation. This structural motif is likely responsible for the enhanced mechanical integrity of these CA monoliths, both before and after activation. Despite being macroporous, the pre-activated CA still exhibits appreciable surface area (~400 m^2^/g) due to microporosity within the carbon ligaments. To increase the accessible surface area in this material, the CA can be thermally activated with carbon dioxide. Thermal activation involves the controlled burn-off of carbon from the network structure in an oxidizing atmosphere resulting in the creation of new micropores as well as opening of closed porosity in the CA framework. Examination of the CAs following activation shows smaller network ligaments relative to the unactivated material, due to burn-off of carbon from the aerogel framework (Figure 9). As expected, the BET surface areas of the activated CAs increase with the length of activation. At shorter activation times, this new porosity is in the form of micropores (pores smaller than 2 nm), as evidenced by the steady increase in micropore volume for materials up to 2,500 m^2^/g (Figure 9).

At longer activation times, however, the micropore volume in these materials drops precipitously. This observation can be attributed to the widening of micropores during activation to sizes that cross the micropore-mesopore boundary, leading to formation of “supermicropores” and/or small mesopores at longer treatment times. While these general trends are similar to those observed in other activated CAs [84,85,86,87], the key difference with these materials is that the larger network features of the pre-activated CA allows access to surface areas in excess of 3,000 m^2^/g, the highest values that we are aware of for a CA. Interestingly, despite the significant mass loss, the activated materials remained monolithic and retained a high degree of mechanical integrity following activation. Due to their hierarchical pore structure and high accessible surface areas as well as their ability to be fabricated as conformable shapes, these materials have potential in a variety of applications, as described in the following sections.

### 3.2. Hydrogen Storage

One area of carbon research that has received significant attention is the use of porous carbon materials as sorbents for hydrogen [88,89,90,91]. Safe and efficient storage of hydrogen is considered one of the main challenges associated with utilization of this fuel source in the transportation sector [92]. An important criterion for effective physisorption is a high surface area that exposes a large number of sorption sites to ad-atom or ad-molecule interaction [93]. Moreover, these sites need to have potential wells that are sufficiently deeper than *kT* if physisorbents are to operate at reasonable engineering temperatures. Porous carbons are promising candidates for hydrogen physisorption due to their lightweight frameworks and high accessible surface areas. The low hydrogen binding energies, however, that are typical of carbonaceous sorbents (~6 kJ/mol H_2_), require that cryogenic temperatures (77 K) be utilized for storage of hydrogen in these materials. In general, the amount of surface excess hydrogen adsorbed on porous carbons at 77 K and ~3.5 MPa varies linearly with BET surface area; gravimetric uptake is ~1 wt % H_2_ per 500 m^2^/g of surface area [89].

**Figure 10:**
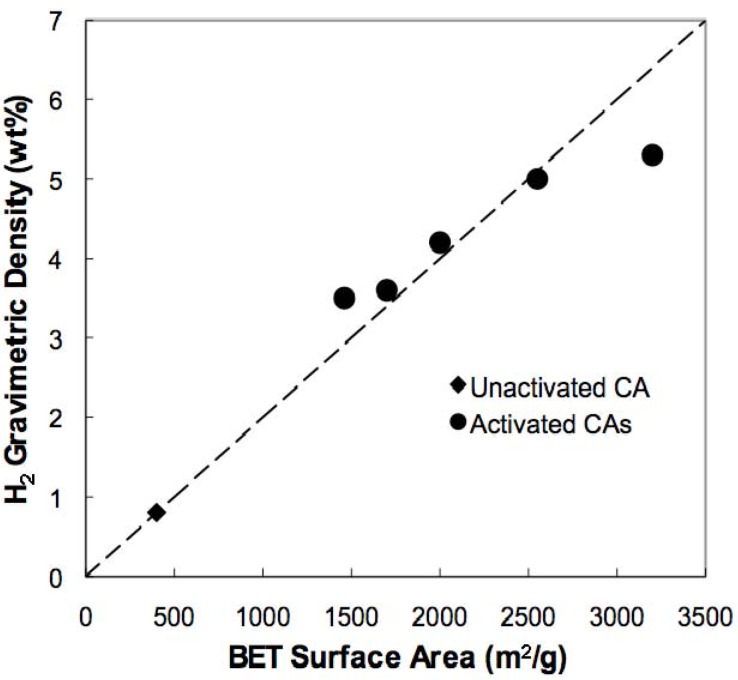
Excess gravimetric density (wt % H_2_) saturation value at 77K as a function of BET surface area. The dotted line shows correlation of 1 wt % per 500 m^2^/g.

We recently reported on the hydrogen sorption properties of our high-surface-area CAs prepared through thermal activation [94]. At 77 K, surface excess H_2_ sorption of these CAs scales with BET surface area up to 2,500 m^2^/g, yielding gravimetric densities up to 5 wt % H_2_ (Figure 10). It is important to note that surface excess hydrogen values are a measure of H_2_ adsorbed on the surface of the CA only and do not account for free hydrogen gas in the pores of the CA. Therefore, total gravimetric hydrogen capacities (free and adsorbed) in these CA materials are higher than the surface excess values. The surface area dependence for activated CAs with surface areas over 3,000 m^2^/g, however, is somewhat weaker, with these materials showing gravimetric densities of ~5.3 wt %. Previous studies have shown that size and shape of the pores in hydrogen physisorbents play a critical role in hydrogen uptake and that the optimal pore structure are slit-shaped pores with diameters between 0.7 and 1 nm [95,96]. As discussed in the previous section, the pore structure in these high surface area CAs changes at longer activation times, leading to a significant loss of micropore volume. This widening of the micropores may be the source of the weakened surface area dependence observed in these materials. Nevertheless, the absolute value of 5.3 wt % is comparable to the highest values measured in porous carbons [89]. In addition to gravimetric capacity, volumetric capacity is an equally important consideration in the design of functional hydrogen sorbents. Depending on the density of the CA, the volumetric capacity of these materials can range from 10 to 29 g H_2_/L. While these values are on a par with those of other porous carbon materials, we are currently investigating methods to optimize the pore structure of the CA sorbents for increased hydrogen energy density.

The hydrogen binding enthalpies measured for the activated CAs were ~ 6 kJ/mol, as would be expected for a carbon-based sorbent. As mentioned above, the low binding energies associated with porous carbons are an obstacle to meeting capacity requirements at reasonable operating temperatures (>273 K). Previous work has shown that hydrogen adsorption energies near 15 kJ/mol, over the full range of surface coverage, are necessary to meet this requirement [97]. Numerous approaches have thus been employed to improve the thermodynamics of hydrogen binding in porous carbons while retaining large surface areas for sorption. The hydrogen spillover effect, for example, has been suggested as a mechanism to increase the reversible hydrogen storage capacities at room temperature in metal-loaded carbon nanostructures [98,99,100]. The spillover process involves the dissociative chemisorption of molecular hydrogen on a supported metal catalyst surface (e.g., platinum or nickel), followed by the diffusion of atomic hydrogen onto the surface of the carbon support. Alternatively, substitutional doping of carbon with boron or other light elements has also been presented as a promising route toward increasing hydrogen binding energy in these sorbent materials [101]. The flexibility associated with CA synthesis allows for the incorporation of such modifiers into the carbon framework. As described in the next section, we have also developed methods that allow for surface modification of these high surface area CA architectures and are currently evaluating the performance of the modified CAs as next generation hydrogen storage materials.

### 3.3. Surface Modifications Using Atomic Layer Deposition

**Figure 11:**
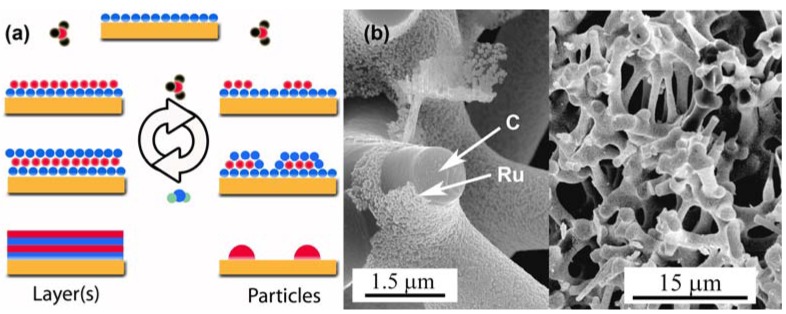
(a) ALD employs sequential, self-limiting surface reactions to overcome diffusion limitations. Both conformal films (left) and individual nanoparticles (right) can be grown, depending on the surface chemistry. (b) SEM micrographs of the fracture surface of a Ru-coated CA at different magnification levels. Carbon ligaments and the Ru nanoparticle film are indicated the arrows labeled “C” and “Ru”, respectively. Part (b) is reproduced from Ref. [106].

Surface modifications of CAs, or nanoporous bulk materials in general, offer yet another means to further add functionalities to these already extremely versatile materials. A good example is the catalytic activity of Pt doped CAs described in the next chapter. Although straightforward in theory, the infusion of metals or metal oxides into nanoporous bulk materials is difficult in reality due to their extremely high aspect ratios. Consequently, techniques commonly use for macro-cellular foams result in inhomogeneous or incomplete coatings. The diffusion limitation can be overcome by employing atomic layer deposition (ALD) which is a special variant of the chemical vapor deposition technique based on a suitable pair of sequential, self-limiting surface reactions (Figure 11a). Consequently, the technique offers excellent atomic level control of the deposited film thickness [102,103,104].

The method can be used for both oxidic and metallic deposits, and generates only volatile co-products thus eliminating the necessity to perform additional reduction and cleaning steps. The morphology of the deposited material depends on the specific surface chemistry, and can range from individual nanoparticles to conformal films thus offering another powerful tool in the design of new nanoporous materials. We employed this technique to deposit W [105], Ru [106], Pt [107], Cu [108], TiO_2_ [109] and ZnO [110] on various aerogel templates, and generally observed good results in terms of uniformity and conformality of the deposits as long as the vapor pressure of the precursor species is sufficiently high. A typical example is shown in Figure 11b which demonstrates the formation of a continuous layer of Ru nanoparticles on the internal surfaces of a CA sample.

### 3.4. Catalysis by Pt doped Carbon Aerogels

Platinum nanoparticle loaded high surface area carbon materials have become the most commonly used cathode catalysts in proton-exchange membrane fuel cells (PEMFCs) and direct methanol fuel cells (DMFCs) [111,112]. However, in order to achieve economic viability, the Pt loading needs to be further reduced to values below 1 mg/cm^2^ [113]. Here, ALD is a promising alternative to traditional techniques such as wet impregnation as it offers precise control of the amount of deposited material.

**Figure 12:**
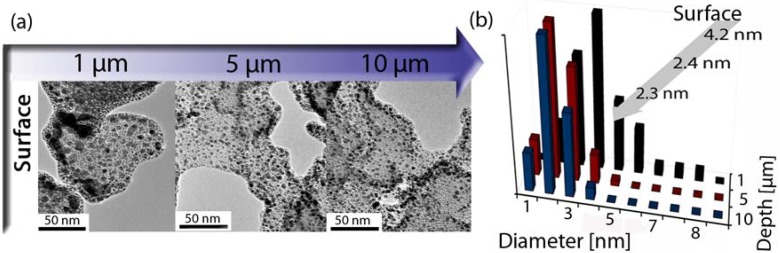
Morphology of a Pt loaded CA (10 ALD cycles). (a) Cross-sectional TEM micrographs revealing the high dispersion of Pt. The images were taken at different depths below the outer surface. (b) Corresponding particle size histogram and averaged particle diameters [107].

We recently tested the catalytic properties of Pt loaded CAs prepared by ALD [107]. The catalytic oxidation of CO with molecular oxygen was chosen as a test reaction [114,115]. The material showed high catalytic activity with a conversion efficiency of nearly 100%, even at loading levels as low as ~0.05 mg Pt/cm^2^. The loading level can easily be controlled by the number of ALD cycles applied, and typical results are shown in Figure 12 (see Ref. [107] for details). The high catalytic activity can be attributed to the high dispersion of Pt on the CA surface. Even for the highest loading levels studied (10 ALD cycles), the Pt particle size stayed well below 5 nm.

**Figure 13:**
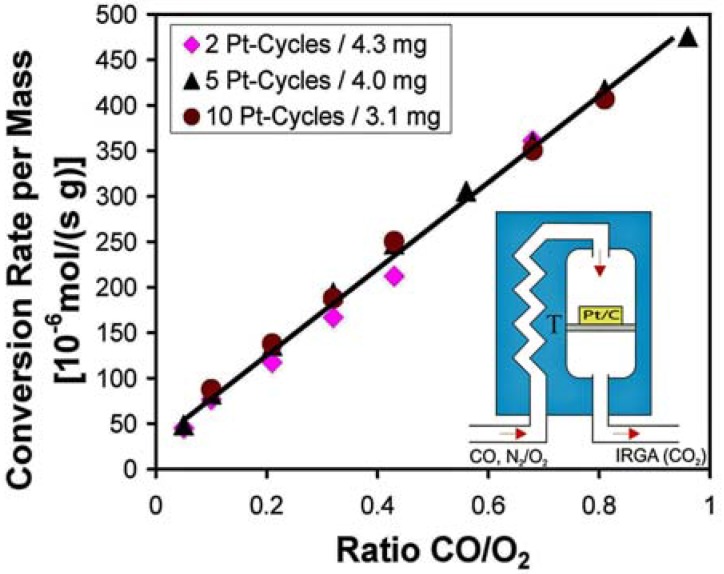
CO conversion rate (normalized with respect to the catalyst mass) as a function of the CO content in the feed for various loading levels. The maximum activity is reached after only two cycles of ALD.

The measurements were carried out using a continuous flow reactor (for a more detailed description see also [107]). Catalytic activity was observed at temperatures of 180 °C and above. At lower temperatures, the Pt catalyst surface is poisoned by CO [115]. The CO conversion rate increases linearly with the CO content in the feed (see Figure 13) thus suggesting that the conversion of CO is close to 100%. Most importantly, it was found that the measured conversion rates were independent of the actual Pt loading, and that the maximum catalytic activity was reached at only two cycles of ALD.

This can be understood in terms of mass transport limitation into the pores. Depending on the assumptions made for mass transport limitations, we calculated turn-over frequencies (TOF) between 4 s^-1^ and 29 s^-1^, in good agreement with model studies on Pt surfaces [115]. The maximum conversion rate is limited by the heat produced by the exothermic CO oxidation which increases the temperature of the catalyst to the point where the CA support is attacked by oxygen. This was only observed for very high conversion rates (~5 × 10^-4^ mol/(s·g)) corresponding to a power production of about 160 W/g.

In summary, ALD is an efficient means to add catalytic activity to an otherwise inert nanoporous support. This allows one to combine the catalytic activity of metal surfaces with the robustness and flexibility offered by CAs.

## 4. Conclusions

The examples discussed above clearly demonstrate that the properties of bulk nanoporous materials are dominated by their surface and interfacial properties. For example, not only the magnitude, but also the sign of the charge induced strain effect of np-Au depends on the actual surface chemistry. The surface chemistry itself, on the other hand, depends strongly on the nanoscale surface morphology and the actual surface composition as demonstrated by the catalytic behaviour of np-Au. Surface chemistry is also an important factor in understanding the stability of these intrinsically unstable materials. Activated CAs are the extreme case of a surface dominated material as they can be best described as 3D surface bulk material. Finally, the example of metal loaded CAs demonstrates that new functionalities can be added by surface engineering. These examples demonstrate that the ability to create new functional materials by controlling the surface and interfacial properties of nanoporous materials holds great promise in the field of sustainable energy applications.
